# Investigation of Zebrafish Embryo Membranes at Epiboly Stage through Electrorotation Technique

**DOI:** 10.3390/membranes13090785

**Published:** 2023-09-09

**Authors:** Aránzazu Sanchis-Otero, María Teresa Reina-Maldonado, José Roldán, Vicenta María Barragán, Sagrario Muñoz

**Affiliations:** 1 Non-Ionizing Radiation Laboratory, National Centre of Environmental Health, Institute of Health Carlos III, 28220 Madrid, Spain; 2 Radiation Group, Department of Signals, Systems and Radiocommunications, Polytechnic University of Madrid, 28040 Madrid, Spain; 3 Department of Structure of Matter, Thermal Physics and Electronic, Faculty of Physics, Complutense University of Madrid (UCM), 28040 Madrid, Spain; vmabarra@ucm.es (V.M.B.); smsm@ucm.es (S.M.)

**Keywords:** zebrafish embryo, electrorotation, membrane permeability, epiboly stage, electrical properties, multi-shell spherical model

## Abstract

A preliminary exploration of the physiology and morphology of the zebrafish embryo (ZFE) during the late-blastula and early-gastrula stages through its electrical properties was performed, applying the electrorotation (ROT) technique. This method, based on induced polarizability at the interfaces, was combined with an analytical spherical shell model to obtain the best fit of empirical data and the desired information, providing a means of understanding the role of different membranes. Suspended in two solutions of low conductivity, the major compartments of the ZFE were electrically characterized, considering morphological data from both observed records and data from the literature. Membrane integrity was also analyzed for dead embryos. The low permeability and relatively high permittivity obtained for the chorion probably reflected both its structural characteristics and external conditions. Reasonable values were derived for perivitelline fluid according to the influx of water that occurs after the fertilization of the oocyte. The so-called yolk membrane, which comprises three different and contiguous layers at the epiboly stage, showed atypical electrical values of the membrane, as did the yolk core with a relatively low permittivity. The internal morphological complexity of the embryo itself could be addressed in future studies by developing an accurate geometric model.

## 1. Introduction

After several decades, the embryo of the zebrafish, *Danio rerio*, has received increasing attention as a relevant alternative vertebrate model to animal experimentation in a wide range of biomedical and fundamental research [1,2]. With an approximate size of one millimeter, its genetic homology to mammals, low breeding and maintenance costs, fast development, with its transparency and optical accessibility up to the larvae stage, make the zebrafish embryo (ZFE) a suitable model for multiple studies [3].

The physiology and morphology of the developing embryo before hatching changes dramatically and quickly during the earliest stages. An overall low water and ion exchange with the external medium, tight junctions between cells in membranes acting as an impermeable barrier to many compounds, and a yolk supplying the nutrients needed for growth and development characterize these stages.

The membranes and layered structures throughout the ZFE body play different functions, such as mechanical and microbial protection of the embryo, the regulation and control of the transport of nutrients and other substances, and even important roles during morphogenetic processes of development [4]. The external, acellular, transparent, tough, and porous shell, called chorion, protects the embryo up to the hatching time and is the first semipermeable barrier of the ZFE to control the passage of compounds depending on their physicochemical properties and their molecular size [5,6]. The chorion encloses the perivitelline viscous fluid containing the developing ZFE, called the perivitelline space (PVS), which is the main barrier to gas and solute diffusion. [7]. The embryo itself, constituting a developing blastodisc, formed from the oocyte cytoplasm streamed toward the animal pole after fertilization, is located over the large yolky spheric sac, sited in the vegetal pole of the activated egg.

Specifically, during the blastula and early-gastrula period, when the basic germ layers and body axes are established through a remarkable transition of the blastodisc from a simple ball of cells to more complex patterns, the propagating waves of Ca^2+^ could play a crucial role [8]. Primarily mediated by this Ca^2+^ signaling ion, the coordination of cell growth and differentiation occurs, and any dysregulation in the signaling cascades of this ion leads to various diseases and disorders [9]. The epiboly stage in ZFE begins between the late-blastula and early-gastrula periods as a key morphogenetic event, extensively studied from cellular and molecular and, more recently, from biomechanical perspectives [10,11]. During epiboly, the developing blastodisc thins and become the blastoderm, which spreads over the yolk sac without changes in volume or shape [11,12].

The morphological modifications during this developmental process, involving the contraction and extension of the extraembryonic membranes, as well as electrophysiological changes in the embryo [13], directly determine its electrical properties (EPs). The biochemical composition and morphological complexity are reflected through capacitance, and ion and metabolite transport in membranes are reflected through conductance. Thus, exploring the dielectric response of the embryo during this developmental stage could provide important and complementary information for the study of epiboly, with applications either for basic developmental studies or for multiple areas of zebrafish research in relation to compound uptake, such as drug discovery, toxicity testing, aquaculture technologies, etc. Among the physical techniques available for investigating the EPs, traditional dielectric impedance spectroscopy (DIS), extensively used with particles in suspension, usually requires complex mathematical expressions for data analysis, taking into account the interacting particles [14]. This drawback has been overcome using the finite element method (FEM) to analyze the electrical response of the sample within a coaxial line, or by applying DIS to individual bioparticles. This is the case for the studies carried out with a suspension of ZFE [15] and with individual medaka fish and frog embryos for the dielectric monitoring of development [16,17], respectively. However, the advantages of studying single biological systems from simpler theoretical models lead to prefer the electrokinetic technique called electrorotation (ROT) for the non-invasive dielectric characterization of tiny bioparticles.

This non-invasive technique, first described in the 1980s [18,19], is the electrically induced torque on a suspended dielectric particle under a rotating electric field. The ROT response or rotation speed of a polarizable biological particle relies on the frequency dependence of its electrical properties (EP), conductivity, and permittivity. The electrical polarization of membranes and structural interfaces determines the ROT spectrum of the particle, characterized by several rotational velocity maxima. The typical ROT spectrum of a single-shell particle describes two peaks during field frequency scanning: the first, at lower frequencies, turning in the opposite direction to the ***E***-field (anti-field), and the second, following the rotating field (co-field) at higher frequencies. In general, the EPs of membranes and bounding membranes are deduced from the anti-field peaks; meanwhile, the co-field peak mainly provides information on the polarization of the inner part of the particle. The theoretical analysis of ROT behavior is usually based on spherical or ellipsoidal multi-shell models or on the superposition of Lorentzian curves. Through these, several ROT studies have demonstrated the potential of their application to embryogenesis studies to differentiate fertilized and unfertilized eggs of vertebrate [20,21] and invertebrate [22] species sized up to 100 μm. Also, millimeter-sized eggs, such as medaka fish embryos, have been successfully used in ROT measurements to investigate dielectric changes during embryonic development [23], overcoming the difficulties previously encountered with the experimental setup for the ROT of axolotl embryos [24].

Thus, employing the same hanging droplet sampling system used with medaka embryos, we developed an experimental system for the ROT of the ZF embryo. In this preliminary study, we present the ROT behavior of ZFE at the epiboly developmental stage maintained in egg water. Electrical polarization at the main interfaces or membranes that make up the embryo is considered through a simplified three-shell spherical model. To obtain additional information, deionized water was used as an external medium for ROT analysis. The influence of membrane integrity on the ROT behavior of embryos is also presented by analyzing the polarizability of embryos exposed to lethal doses of ethanol.

The results confirm the combination of experimental electrorotation data and an analytic embryo model as an adequate tool to derive the dielectric properties of the principal compartments of the ZF embryo. The dielectric properties obtained for each structural compartment are discussed and compared to the results obtained from the ROT of medaka eggs. Although some mentioned studies have already analyzed changes in the dielectric properties during embryogenesis in different species [17,21,23] or have even applied DIS to analyze ZFE membrane permeability during cryoprotectant treatment [25], to our knowledge, not much experimental work appears to have specifically addressed the dielectric characterization of the zebrafish embryo. The electrical characterization addressed in this work, at the epiboly stage of the ZFE, could also shed light on new studies using transient exposure protocols to ethanol [26] or other multiple toxic agents at this stage to investigate morphological and physiological changes. Moreover, the electrical characterization of ZFE may provide a key aspect for a deeper understanding when assessing the possible biological effects of electromagnetic fields investigated in this relevant alternative model [27,28].

## 2. Materials and Methods

### 2.1. Polarization and Rotation of Eggs

We only present the basic assumptions and equations to explain the physics behind the electrorotation technique. The theory about ROT can be found in detail elsewhere [29,30,31]. The electrical polarization of the ZF embryo resulted from the charge distribution induced by an external electric field ***E***, leading to an induced dipole moment ***p***. This free charge accumulated at the interfaces between compartments with different electrical properties, and the permanent dipoles reoriented themselves, both contributing to the induced dipole. Since a finite time was required to build up the surface charge at the interfaces, a phase shift appeared between the rotating AC ***E***-field and ***p***, and the mechanical effect of rotation appeared. The induced dipole moment is the function of the ***E***-field through the polarizability *α* of the particle, ***p*** = *α**E***. Polarizability provides a measure of the strength of the effective dipole moment as a function of the EPs, permittivity *ε*, and conductivity *σ*, of the different media involved. These determine the response to the applied ***E***-field, storing or losing energy, respectively, from *ε* and *σ*. Both electrical parameters are related to the frequency-dependent complex permittivity, $\varepsilon^{*},$ as follows:

(1) $$\varepsilon^{*}\left(f\right)=\varepsilon -j\frac{\sigma }{2\pi f}$$

where *j* = (−1)^½^. Referring to the case of a heterogeneous particle suspended in a liquid medium, the effective polarizability $a_{eff}$ results from the relationship between the complex effective permittivity of the particle $\varepsilon_{eff}^{*},\;$and the complex permittivity of the external medium $\varepsilon_{ext}^{*}$:

(2) $$\alpha \;(f)=4\pi \varepsilon_{ext}Im\left(\;\frac{\varepsilon_{eff}^{*}-{\varepsilon_{ext}^{*}}_{\;}}{\varepsilon_{eff}^{*}+2\varepsilon_{ext}^{*}}\right)$$

where “*Im*” refers to the “imaginary part of”, indicating the out-of-phase response to the ***E***-field. The frequency-dependent expression for the rotational velocity Ω is given by:

(3) $$\Omega \left(f\right)=\frac{1}{8\pi \eta }Im\left(\alpha_{eff}\right)E^{2}=\frac{\varepsilon_{ext}}{2\eta }Im\left(\;\frac{\varepsilon_{eff}^{*}-{\varepsilon_{ext}^{*}}_{\;}}{\varepsilon_{eff}^{*}+2\varepsilon_{ext}^{*}}\right)E^{2},$$

where *η* is the medium viscosity. Then, the frequency-dependent rotation rates of the ZFE described a spectrum of experimental data according to its complex effective permittivity. The rotational movement, either co-field or anti-field, could be observed, including several maxima or minima along the frequency range. Those frequency peaks might correspond to the different relaxation processes at the interfaces, characterizing the restoration of the system disturbed by the ***E***-field to a new equilibrium configuration.

### 2.2. ROT Chamber

The electrorotation system consisted of an eight-planar-electrode chip fabricated over a glass substrate and two printed circuit boards (PCB) to carry the electrical signals. The electrode chip was fabricated over a clean glass substrate where, firstly, the designed pattern of 8 electrode tips was arranged around a 1.25 mm diameter gap, previously reproduced by the standard photolithography process, and then two layers of metals were deposited by Joule effect evaporation (Cr/Au, 10 nm/140 nm in thickness, respectively).

**Figure 1:**
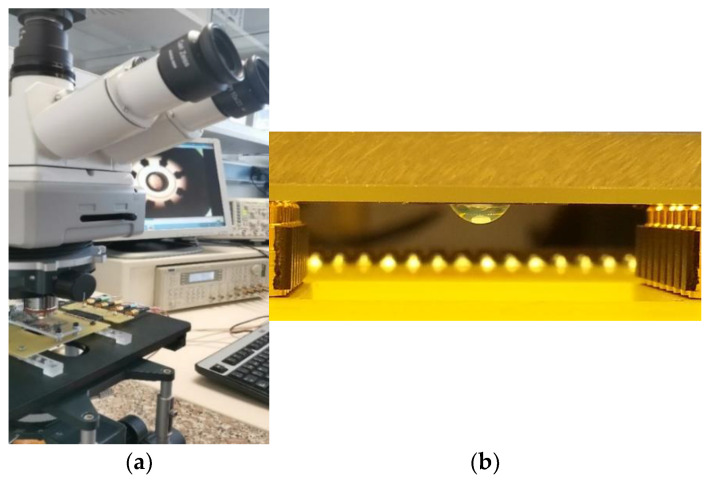
(**a**) Experimental setup for electrorotational observation of zebrafish embryos; (**b**) Hanging droplet with the embryo under electrorotation.

A PCB was designed to host glass chips of 30 mm × 26 mm in size, allowing electrical connections through two parallel series of pogo pins. These pogo-pin connectors were disposed symmetrically on two of the interior edges of a drilled rectangular hole in the PCB, creating an opening for microscope observation of the ROT-electrode chip. In this ROT chamber, the chip was placed upside-down to match pads to pogo pins and was fastened through a methyl methacrylate layer (MMA), allowing the sample to be observed in a hanging droplet, as shown in Figure 1. The ROT chamber was easily inserted and removed from the assembly, both for sample preparation prior to testing and for cleaning the chip. The second PCB, containing four input ports, was the stage driving the signal from the generator to the PCB housing the chip. The driving PCB was plugged using pin connectors to the first PCB, making assembly quick and easy.

### 2.3. Zebrafish Embryos Collection, Maintenance and Exposure

Zebrafish embryos obtained from wild-type zebrafish progenitors originating from a local pet store were maintained at standard conditions for breeding at the Fishes laboratory of the National Centre of Environmental Health. For fish husbandry, two males and one female per small breeding tank were placed together overnight. According to the photoperiod established (14/10 h dark/light), after the onset of room light, the females started to lay some eggs while the males fertilized them. Afterward, zebrafish embryos were collected from the tanks and were deposited into glass beakers with egg water. The medium to maintain the developing embryos was prepared following UNE-EN ISO 7346-3: 1998. The debris was removed, and the eggs were washed several times with egg water, examining their viability under the stereo microscope. For ROT measurements, viable eggs were selected, considering similar developmental stages, and disposed individually into the wells of a plate fulfilled with a medium conductivity close to 200 μS/cm (FE30 FiveEasy™, Mettler-Toledo GmbH, Greifensee, Switzerland) obtained from the dilution (1:4) of the egg water. The plate was then kept in a water bath at 26.5 ± 0.5 °C, resulting in a temperature of 25.2 ± 0.5 °C in each well, slowing down the rate of development according to the Kimmel scheme foreseen at 28.5 ± 0.5 °C [12]. Non-viable embryos for ROT experiments were obtained from viable eggs exposed to lethal doses of ethanol.

### 2.4. Electrorotational (ROT) Assays

The ROT response of the zebrafish embryos suspended in egg water consisted of a frequency-dependent rotational movement arising from the different embryo and medium polarizabilities. The embryo was then induced to rotate in a co-field or anti-field direction, exhibiting characteristic frequencies when the rotation was faster (peak frequencies) and describing a spectrum of experimental data. A low-ionic media conductivity of 1.15–200 µS/cm was used for this study.

For the ROT assay, firstly, a drop of 12 µL of egg water was placed over the microelectrode gap, and secondly, the selected sample from the well plate was collected and carefully pipetted into the drop of water [23]. When other conductivities were used for ROT, the embryo was pre-washed by immersing it in a beaker filled with that medium, and the ROT test was performed sequentially with the same sample but was suspended in the new test medium. Once the embryo was arranged, the PCB containing the electrode chamber was connected to the PCB driving signal, leaving the embryo in a hanging droplet, as described in Section 2.1 (Figure 1b). Four 90° phase-shifted sinusoidal signals were applied from the generator TTi TGA12104 (Thurlby Thandar Instruments Ltd., Huntingdon, UK) to the eight electrodes, short-circuited pairwise, with a 3.0–7.5 V_pp_ amplitude, and decreased the frequency range from 20 MHz to 1 kHz with five values per decade. The sample was observed through a right microscope (Axio Examiner A.1, Karl Zeiss, Oberkochen, Germany) using a magnification 2.5× objective; meanwhile, the attached video camera (Hitachi VK-C150) registered the rotational motion. By post-processing the recorded videos, we obtained normalized ROT spectra with respect to the *E_rms_* field strength squared for comparison among different voltage inputs.

### 2.5. Dielectric Modelling of Zebrafish Embryos

#### Multi-Layer Spherical Model

A multilayered spherical model was used as a simplified approach for the theoretical analysis of the ROT behavior of the ZFE. Following the *smeared-out* approach proposed by Huang [32], a heterogeneous spherical particle can be described as a homogeneous one. Sequentially, adjoining compartments, denoted by *i*, *i* + 1, *…* from the inside out, were replaced by a sphere of radius *R_i_*_+1_ with effective, frequency-dependent complex permittivity $\varepsilon_{(i+1)eff}^{*}$ equivalent for *i +* 1 dielectric media, where *i* = 1, …, *N.* The equivalent complex permittivity $\varepsilon_{eff}^{*}\;$of the *N*-shelled spherical particle, with *N* + 1 dielectric media, is given by the expression:

(4) $$\varepsilon_{eff}^{*}\left(f\right)=\varepsilon_{(N+1)\;eff}^{*}\left(f\right)=\varepsilon_{(N+1)\;}^{*}\left\{\frac{{\left(\frac{R_{N+1}}{R_{N}}\right)}^{3}+2\left(\frac{\varepsilon_{N\;eff}^{*}-\varepsilon_{N+1}^{*}}{\varepsilon_{N\;eff}^{*}+2\varepsilon_{N+1}^{*}}\right)}{{\left(\frac{R_{N+1}}{R_{N}}\right)}^{3}-\left(\frac{\varepsilon_{N\;eff}^{*}-\varepsilon_{N+1}^{*}}{\varepsilon_{N\;eff}^{*}+2\varepsilon_{N+1}^{*}}\right)}\right\}$$

**Figure 2:**
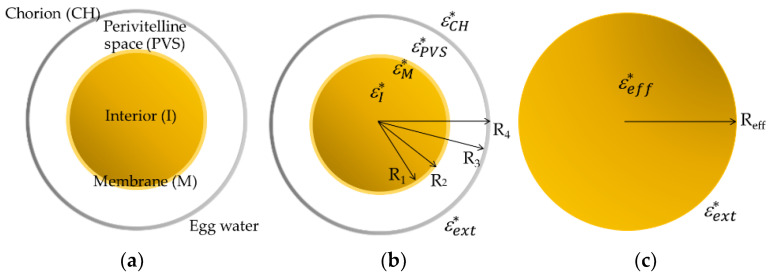
(**a**) The millimeter-sized zebrafish embryo is simplified and represented by a three-shell model (TSM) with three concentric layers enveloping the interior (I): the membrane (M), the perivitelline space (PVS) and the chorion (CH). (**b**) Each compartment of the multilayered spherical model is characterized by its frequency-dependent complex permittivity $\varepsilon_{}^{*}$, and its corresponding geometrical dimension: *R*_1_ is the radius of the yolk core; and *R*_2_–*R*_1_, *R*_3_–*R*_2_ and *R*_4_–*R_3_* determine the M, PVS and CH thicknesses, respectively. (**c**) The heterogeneous dielectric structure assumed for the ZFE and illustrated in (**b**) is replaced by a homogenous dielectric sphere characterized by an equivalent effective complex permittivity $\varepsilon_{eff}^{*}$ and a total radious *R_eff_* (=*R_4_*), suspended in an aqueous medium of permittivity $\varepsilon_{ext}^{*}$.

Correspondingly, Figure 2 illustrates the three-shell model (TSM) assumed for the ZFE, made up of four main concentric spherical structures, where the blastodisc and the yolk sac, considered as one and referred to as the interior (I), are surrounded by three layers: a membrane (M), the perivitelline space (PVS) and the chorion (CH). Both embryo and PVS dimensions were established directly by measuring the images obtained from the samples, while both egg membrane and chorion thicknesses were obtained from the literature [33,34].

The ROT response obtained for several ZFE at the epiboly stage in egg water presents two anti-field peaks at the lower part of the spectrum and points to having a co-field peak beyond the experimental limit frequency (20 MHz) and closely preceded by a slight dispersion around 6 MHz. Since the number of these peaks and dispersions is related to the number of concentric dielectric interfaces within the biological particle, only observable in some experimental cases [35,36,37], we used a simplified three-layer spherical model (four interfaces) for the analysis of the experimental data.

## 3. Results

### 3.1. ROT Spectra and Dielectric Characterization of ZFE

When epiboly, or the spreading and extension of the blastodisc over the yolk cell, reaches approximately the same percentage among the ZFE, those selected were pipetted sequentially to the chamber for ROT assay; each of them was first in a 190 µS/cm conductivity medium, and then in deionized water of 1.15 µS/cm. In this preliminary study, only embryos presenting a similar percentage at the epiboly stage and ROT behavior in egg water were included in the ROT analysis.

**Figure 3:**
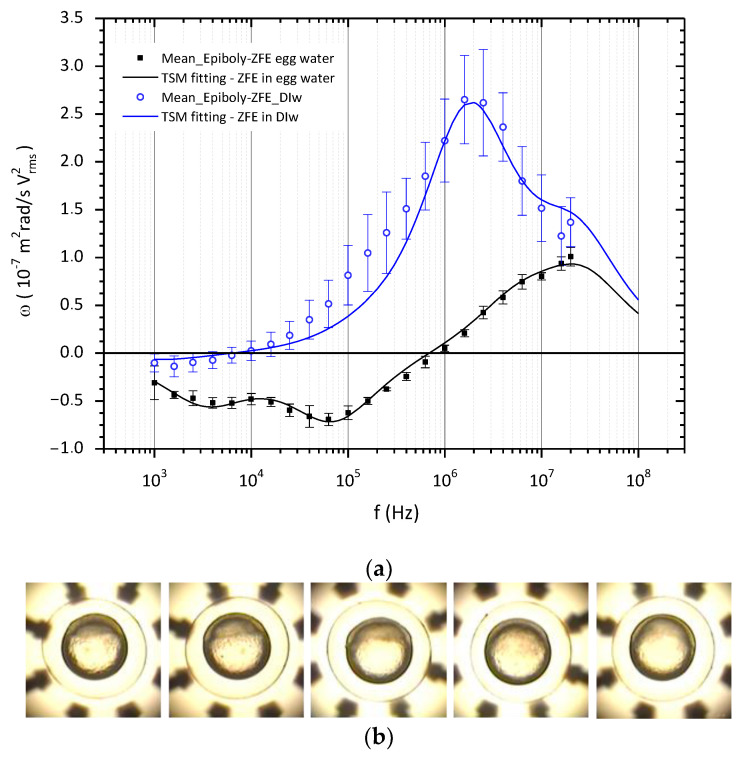
(**a**) ROT spectra and fitting curves of ZFE suspended in a media of conductivity: 190 µS/cm—continuous black line—and DI water (1.15 µS/cm)—dotted blue line. (**b**) ZFE at epiboly stage under ROT in the chamber.

As expected, the ROT spectra of ZFE in media of different conductivities present different characteristic profiles, as shown in Figure 3. Embryos suspended in 190 µS/cm exhibited a distinctive ROT behavior in the lower frequency region, depicting two soft peaks around 4 kHz and 60 kHz; meanwhile, the rotation speed in the co-field region increased continuously with frequency but did not reach a maximum before the experimental limiting frequency of 20 MHz, although it presented a slight dispersion at 10 MHz. In deionized water (DIw), the small anti-***E*** region of the spectra presented almost no response, but a pronounced co-field behavior was observed from 10 kHz, reaching a maximum velocity close to 2 MHz. The differences in the standard deviation of the ZFE ROT in both conductivity media, bigger for DIw, indicate that the behavior of embryos in DIw is much less uniform than in egg water, as is to be expected given that the eggs were suspended in a medium of lower conductivity. It is likely that different processes of ion exchange happen in each ZFE to adapt to the new external conductivity.

Considering the model described in Figure 2, the TSM was used to extract the electrical properties of each compartment of the embryo when the estimated response fit the experimental ROT data. For better accuracy of electrical characterization, a scaling factor, *sf*, was introduced, considering the square of the *E_rms_* field, the friction of the egg within the hanging droplet, and the medium viscosity *η*, taking values from 2.5 × 10^11^ to 3.5 × 10^11^. The matched curves, as shown in Figure 3, resulted from the variation in the EPs of each compartment once fixed in the external medium and the structural dimensions of the embryos, taking as reference values those published for medaka fish eggs [23]. The electrical characterization of the ZFE at the epiboly stage, obtained by applying Equation (4) for the TSM model, is displayed in Table 1.

As previously stated, the anti-E region of ROT spectra is related to the membranes and bounding membranes: chorion and core membrane. According to the interpretation of the ROT of protoplasts made by Fuhr [36], a multi-shell system can be separated into single-shell spheres to understand the entire system’s behavior. That study showed that the polarization of the outer shell—the chorion- was responsible for the first peak; meanwhile, the second peak was due to the electrical properties of the third compartment—the vitelline membrane. The high permittivity of 90 for the outer chorionic membrane, the embryo’s first protective barrier, could be due more to its numerous pores than to its macromolecular composition. Moreover, this multilayered structure, mainly composed of glycosylated and non-glycosylated proteins, is characterized by a negative charge to which positive ions can be attached [33,38]. These polar and porous natures might then explain this permittivity value. The relative low conductivity values of chorion are consistent with the role of the leakage membrane in low conductivity external media, allowing water and electrolytes to penetrate PVS [39] but blocking the passage of compounds depending on their molecular size and physicochemical properties. In deionized water, the polarizability of the embryo is mainly dominated by the inner structures, so the weak influence of the chorion in the co-field region of the spectrum allows small variations in its conductivity value.

The strong influence of perivitelline space conductivity on the ROT behavior of the ZFE can be clearly seen throughout this study. The permittivity of the perivitelline fluid equaled that of water, as can be deduced from the genesis of the PVS. When the PVS formed, the oocytes activated were immersed in egg water of high conductivity, close to 800 μS/cm. This water entered the oocyte to form the perivitelline fluid, and then, the embryos arranged for ROT assays were kept in diluted (1:4) egg water for more than two hours. So, this process could well explain the value of 300 μS/cm estimated for the PVS from ROT in egg water. Furthermore, in accordance with the role of chorion in water and electrolyte exchange, the ROT behavior of embryos in deionized water—as shown in Figure 3 and following the estimated EPs in Table 1—shows a clear dependence on an expected drop in the conductivity of the PVS fluid. This may be accompanied by a slight increase in the conductivity of the chorion.

With respect to what could be expected for a biological membrane, a relatively high dielectric constant of 50 and a conductivity of 0.1 µS/cm have been estimated for the membrane from our ROT experiments. However, this simplified model assumes as a single entity three different and contiguous membrane layers: the enveloping layer (EVL), the external yolk syncytial layer (E-YSL), and the yolk cytoplasmic layer (YCL). The EVL is a monolayer of flatted cells on the top of the blastodisc, which describes a ridge-like pattern on the external surface [34] and represents a low permeable interface with the PVS [7,40]. Next, the E-YSL, with contractile activity at this epiboly stage, also presents a highly convoluted surface. Moreover, as a syncytium, defined as *a mass of cytoplasm containing many nuclei and enclosed in a cell membrane*, ions, metabolites, and molecules can diffuse freely [7]. Adjacent to the E-YSL, the YCL surrounds the dense yolk core, which contains longitudinally organized microtubules [41], giving the YCL a polar nature. From this description, it can be understood that the folding, roughness, syncytial nature, and presence of polar elements could explain the high value obtained for the permittivity of this surface layer, which is supposed to be the embryo membrane. Meanwhile, as expected, the low conductivity value of 1 × 10^−7^ S/m was in line with the low ionic permeability role of a membrane that allowed ZFE to grow comfortably in media with different ionic concentrations, acting as a selective barrier.

Finally, the relative permittivity, between 15 and 20, and a conductivity of 0.2 S/m were estimated for the interior of these ZF embryos from the ROT curve. These electrical values, obtained through the TSM model, should be explained since the inner complexity of the ZFE interior mainly governs the high-frequency ROT response. In the epiboly phase, not only do the animal and vegetal poles coexist within the core, but also the blastodisc narrows to form the blastoderm, which, under the push of the large volume of yolk, gradually spreads out until it completely engulfs the yolk mass by the end of epiboly. The yolk cell is mainly composed of phospholipoproteins [12], which could explain the low value of permittivity; meanwhile, the deep cells of the blastoderm, intercommunicated through the intracellular membranes or by extracellular space between cells [42], could be responsible for the conductivity value. When the ROT response was observed in deionized water, it was seen that the estimated values for both membrane and interior PEs remained unchanged. This result is reasonable since the membrane is considered a selectively permeable barrier, as explained before.

### 3.2. ROT Spectra and Dielectric Characterization of Non-Viable ZFE

**Figure 4:**
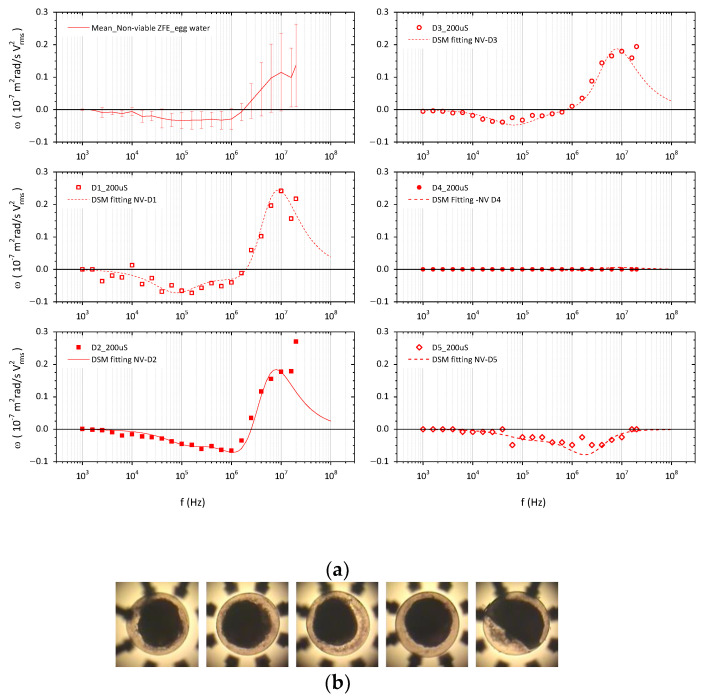
(**a**) ROT spectra and fitting curves of dead ZFE in egg water (190 µS/cm). From top to bottom and from left to right, the graphs show first the mean ROT response of non-viable embryos and then the experimental ROT data of embryos D1 to D5, each with the curve fit obtained from a double-shell spherical model (DSM). (**b**) The non-viable coagulated embryos under ROT in the chamber.

The ROT behavior of dead embryos after lethal exposure to ethanol is shown in Figure 4. The variability of ROT responses, especially at higher frequencies, led us to analyze them individually for a better comprehension of the associated changes in their EPs. The ethanol at this high concentration completely dehydrated the embryo and denaturalized the proteins, making the yolk membrane and the interior it enclosed coagulate and appear as an irregular and inseparable structure. Then, considering the morphological changes induced by this chemical exposure, the electrical characterization of each dead embryo in 190 µS/cm egg medium was approached using the double-shell spherical model (DSM), and the obtained data are summarized in Table 2.

Ethanol is soluble in an aqueous medium and easily passes through the chorion and biological membranes. As shown in Figure 4, ethanol-treated embryos presented a drop in their ROT response, corresponding to a general decrease in ZFE polarizability. The data obtained for the chorion suggests that it still behaves as a semipermeable barrier with a subtle increase in conductivity. But, when no rotational movement was observed, as in the case of embryo D4, there was a significant increase in chorion conductivity, up to 30 mS/m, together with a higher decrease in PVS permittivity, probably due to a higher ethanol influx in the PVS. The resultant decrease in the EPs obtained for this compartment—with *ε_r_* between 30 to 54 and *σ* between 5 and 7 mS/m—was consistent with the influx of ethanol and its electrical properties: a dielectric constant of 25.3 and a conductivity of 0.063 mS/m. Equally, the EPs for the coagulated core were in accordance with the effects of the solvent, keeping the values for the dielectric constant was, and lowering the conductivity—ranging from 6 to 13 and from 5 to 18 mS/m, respectively—correspondingly to the electrical values of dehydrated biological tissues and non-viable organisms.

## 4. Discussion

The EPs of zebrafish embryos at the epiboly stage from their ROT behavior have been investigated using a three-shell spherical (TSM) model to obtain the best fit of ROT spectra. As shown before in the literature with medaka [23], the electrorotation technique can be successfully applied to manipulate mm-sized bioparticles and electrically characterize them through the corresponding spherical model. According to our results for ZFE, some differences were found between both teleost species, reflecting their intrinsic structural variations and the different stages of embryonic development considered in each case. The observed influence of the external medium conductivity on the chorion and the PVS conductivities was in accordance with the direct dependence found in the case of medaka embryos, although in this study, no linear relation was assumed. Moreover, the obtained EPs for these compartments were quite different from those extracted for the medaka, which followed the expressions *σ_CH_ =* 0.5 *σ_ext_* and *σ_PVS_ =* 0.3 + 30 *σ_ext_* (S/m). With a ZFE chorion around 20% thinner, the conductance of this outer envelope was around 86% lower than that extracted for medaka, considering our experimental conditions with *σ_ext_* = 19 mS/m, and its dielectric constant was three times higher. An attempt could be made to explain these discrepancies by looking at the chorion structures, which in both species consisted of three distinct zones: the inner, middle, and outer layers. However, as described in the literature, the ZFE chorion, derived solely from the primary envelope of the oocyte, presents multiple open pore canals that penetrate the inner two zones and an outer surface covered with dome-like projections [34,38]. Meanwhile, in medaka, these inner layers are enclosed in a filamentous coat originating from the secondary envelope of the oocyte [43] without apparent pore canals, and the outer layer is covered with attached and non-attached filaments [44]. Furthermore, the fact that the permeability of the chorion was regulated by the concentration of cations in the culture medium, such as Ca^2+^ and Na^+^ [45], might explain the egg envelope’s lower conductivity and higher permittivity in zebrafish, considering different water hardness in the solutions, which were prepared to maintain both embryos’ species. As for PVS, which was thicker in the ZFE, under our experimental conditions, its *σ_PVS_* was only 3.4% that of the medaka embryos. It is difficult to explain this substantial difference, considering that both are freshwater fishes, have water- and electrolyte-permeable chorions, and are maintained in low conductivity media, leading us to consider that the high conductivity value assumed for the PVS of medaka might be incorrect. The value of 4.43 mF/m^2^ found for the specific capacitance of the yolk membrane is consistent with the capacitance *C_ym_* = 4–5 mF/m^2^ reported for medaka embryos, where its conductivity is quite different, as well as the EPs of the yolk core, probably due to the different developmental stage considered and the complexity of this compartment of ZFE at the epiboly stage.

## 5. Conclusions

The combination of analytical shell spherical models and an experimental ROT chamber has shown electrorotation to be a useful tool when obtaining insights into the role of zebrafish embryo membranes at the epiboly stage. The dielectric characterization in this key morphogenetic period from ROT experiments in two different conductivity media was performed and compared to the ROT response of dead embryos. From our analysis, the relative high permittivity and low conductivity extracted for the chorion might reflect both its porous structure and its role as a semipermeable barrier. The observed strong influence of the perivitelline fluid on ROT behavior with the change in the external medium is in accordance with the diffusion functions of this space. The considered membrane of the egg presented a relatively high dielectric constant, probably related to the real structural complexity and functionality of what is modeled simply as a biological membrane. However, the obtained low conductivity is in accordance with the selective barrier characteristic of biological membranes. The interior also simplified through the analytical model, presents a low permittivity value with respect to the typical cell interior, which could be perfectly justified by the important lipid content of the yolk mass. Meanwhile, the developing cells of the embryo over the yolk contribute to the value conductivity obtained for the ZFE interior.

Although these results point out the capability of the proposed setup system to detect the dielectric permittivity and conductivity of different layers, some experimental and theoretical improvements were needed to yield valuable information from the developing embryo. On the one hand, complementary electrokinetic parameters, such as the crossover frequency or higher experimental frequencies to reach the co-field ROT frequency peak, could provide important information for characterizing the complex interior. On the other, a numerical model mimicking morphological changes in the main membranes of the developing embryo during the late-blastula and early-gastrula period might improve the accuracy of this preliminary study and obtain a deeper understanding of the role of ZFE complex structures in its ROT response. Then, the ROT technique might be more efficiently applied for developmental and toxicological studies through the determination of dielectric properties’ changes at this early stage, as well as to assess other chemical or physical environmental factors on this important biological model. Especially and specifically, the ZFE dielectric characterization has a potential and important application for addressing the assessment of possible biological effects from exposure to electromagnetic fields.

## Figures and Tables

**Table 1:** The EPs of the main compartmental structures in ZFE at the epiboly stage suspended in 190 µS/cm egg water and DI water (1.15 µS/cm) media. The geometrical parameters measured, thickness (*δ*) and radius (*r*), were measured on average in the case of PVS thickness and the total diameter of the ZFE embryos; meanwhile, structural parameters for chorion and egg core membrane were found in the literature.

	Parameters	External Medium	Chorion	PVS	Membrane	Interior
	*δ* or *r* (μm)	-	3	210	0.1	397
Medium 1	*ε_r_*	78	90	78	50	15
	*σ* (mS/m)	19	0.25	30	1 × 10^−4^	200
Medium 2	*ε_r_*	78	90	78	50	15
	*σ* (mS/m)	0.115	0.35 ± 0.1	15	1 × 10^−4^	200

**Table 2:** The EPs of the resulting compartmental structures in dead ZFE suspended in egg water, with *ε_r_* = 78 and *σ* =190 µS/cm. The thickness (*δ*) and radius (*r*) of each ZFE has been measured, assuming layers in concentric spheres. Thus, in all cases, *δ_CH_* = 3 μm, and dead embryos D1 and D3: *δ_PVS_* = 170.8 μm, *r_INT_*= 439.2 μm; D2 and D4: *δ_PVS_* = 146.4 μm, *r_INT_*= 463.6 μm; and D5: *δ_PVS_* = 195.2 μm, *r_INT_*= 439.2 μm.

NV ZFE in Egg Water		Chorion	Perivitelline Space	Egg Core
D1	*ε_r_*	90	40	10
	*σ* (mS/m)	0.35 ± 0.05	6	18
D2	*ε_r_*	90	53.5 ± 0.5	7
	*σ* (mS/m)	0.55 ± 0.05	6	14
D3	*ε_r_*	90	29 ± 1	7
	*σ* (mS/m)	0.32 ± 0.02	5	11.5 ± 0.5
D4	*ε_r_*	90	35	13
	*σ* (mS/m)	30	6.5	5
D5	*ε_r_*	90	53	6.5 ± 0.5
	*σ* (mS/m)	0.55 ± 0.05	6	**7**

## Data Availability

Not applicable.
